# Strongly-coupled plasmas formed from laser-heated solids

**DOI:** 10.1038/srep15693

**Published:** 2015-10-27

**Authors:** M. Lyon, S. D. Bergeson, G. Hart, M. S. Murillo

**Affiliations:** 1 Joint Quantum Institute and Department of Physics, University of Maryland, College Park, Maryland 20742, USA; 2 Department of Physics and Astronomy, Brigham Young University, Provo, UT 84602, USA; 3 New Mexico Consortium, Los Alamos, NM.

## Abstract

We present an analysis of ion temperatures in laser-produced plasmas formed from solids with different initial lattice structures. We show that the equilibrium ion temperature is limited by a mismatch between the initial crystallographic configuration and the close-packed configuration of a strongly-coupled plasma, similar to experiments in ultracold neutral plasmas. We propose experiments to demonstrate and exploit this crystallographic heating in order to produce a strongly coupled plasma with a coupling parameter of several hundred.

Strong coupling occurs when the inter-particle interaction energy in a system exceeds the random thermal energy. Strong coupling occurs in fields as diverse as quark-gluon plasmas^1^, ultracold atoms in the BEC-BCS crossover^2^,^3^, interactions at extremely high energy density^4^, quantum dots^5^, superconductivity^6^, and ultracold neutral plasmas^7^. When a system transitions into a regime in which the inter-particle interactions form the dominant energy scale, new physical effects can occur.

In plasma physics, strong coupling is predicted to have significant consequences. Strong coupling leads to long-range spatial ordering, causing the plasma to look less like an ionized gas and more like a Coulomb liquid. Long-range spatial order leads to cancelations in nearest-neighbor force terms. Consequently, ordering plays the role of shielding, leading to the paradoxical conclusion that ions of a given energy can approach one another more closely when the plasma is strongly coupled compared to the case when no order is present^8^,^9^,^10^,^11^. The effects of spatial ordering can enhance ion collision rates *exponentially*^12^,^13^,^14^,^15^.

In plasma physics, strong coupling is difficult to achieve. Strong (Coulomb) coupling is defined as the ratio of nearest-neighbor electrical potential energy to the kinetic energy,


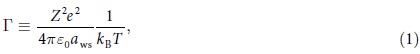


where *Z* is the ionization state, *a*_ws_ = (3/4*πn*)^1/3^ is the Wigner-Seitz radius, and *n* is the ion density. Notice that strong coupling, when Γ > 1, occurs for high *Z*, high density, and/or low temperature. Ionization typically occurs when kinetic energies exceed atomic ionization energies. The corresponding high-speed Coulomb collisions are relatively brief, infrequent, and long-range. The kinetic energy is high in most plasmas, resulting in low values of Γ ≪ 1. It is possible that strong coupling is achieved in high-energy-density plasmas^16^. However, determining the plasma parameters to high precision can be challenging in these rapidly changing complex environments.

One barrier to achieving strong coupling in plasmas is called disorder-induced heating (DIH). This effect has been characterized in a number of ultracold neutral plasma (UNP) experiments^3^,^17^,^18^,^19^,^20^. In those experiments, laser-cooled atoms are resonantly ionized. As the plasma evolves, the rms ion velocity is measured directly. Although the plasmas are formed from essentially zero-temperature atoms, the initially non-interacting atoms suddenly experience the repulsive Coulomb interaction after ionization. The ions move to minimize their nearest-neighbor potential energy, and the electrical potential energy in the disordered system is converted to thermal energy. In these UNP experiments, the ion temperature is determined by the disorder in the initial system. The DIH process is rich in physics, as non-equilibrium dynamics can play a strong role in plasma evolution^21^.

This disorder-induced heating barrier could be overcome by ionizing a “pre-ordered” system. In laser-cooled gases, significant efforts are underway to do this using higher ionization states^22^, the Rydberg excitation blockade^23^,^24^,^25^, adiabatic expansion^26^, and other methods. However, avoiding DIH requires the spatial order in the initial system to match exactly the order that will exist in the strongly-coupled plasma state.

Disorder-induced heating can be a problem even in high density, high temperature plasmas^18^. In this paper we show that a form of DIH occurs in laser-produced plasmas when the crystallographic structure of the initial system does not overlap the close-packed configuration of a strongly coupled plasma^27^,^28^. Strictly speaking, this is not disorder-induced heating per se, but rather heating that arises when the pair distribution function in a given crystal type mismatches the close-packed pair distribution function in a strongly-coupled plasma. Even though the initial lattice may be highly ordered, it is “disordered” relative to the strongly-coupled plasma state. We call this heating effect crystal mismatch heating (CMH). We show that high energy-density plasmas can be strongly coupled if the initial system is appropriately chosen. We calculate the heating that results when different crystallographic configurations are impulsively ionized. For an appropriately chosen initial system, the value of the strong coupling parameter Γ should be high enough to observe Wigner crystallization in the plasma state.

## An Example: a Strongly-Coupled Graphite Plasma

In ref. 29, Brown *et al.* describe an impressive experiment at the Linac Coherent Light Source in strongly-driven carbon. A thin graphite foil was illuminated with a short optical laser pulse. The laser pulse compressed and heated the sample to densities on the order of 1 to 2 × 10^23^ cm^−3^ and an estimated ion temperature of 5,000–10,000 K. The ion charge state was estimated to be *Z* = 4.5. Under these conditions, Eq. 1 gives Γ = 500. A highly-ordered Coulomb crystal was expected to form^30^. At a variable time after the optical pulse, a free electron laser x-ray pulse illuminated the melted carbon sample. The crystal structure of the sample was investigated using x-ray scattering^31^. The scattering data indicated that the plasma was in a partially correlated state but without any evidence of Wigner crystallization.

**Figure 1:**
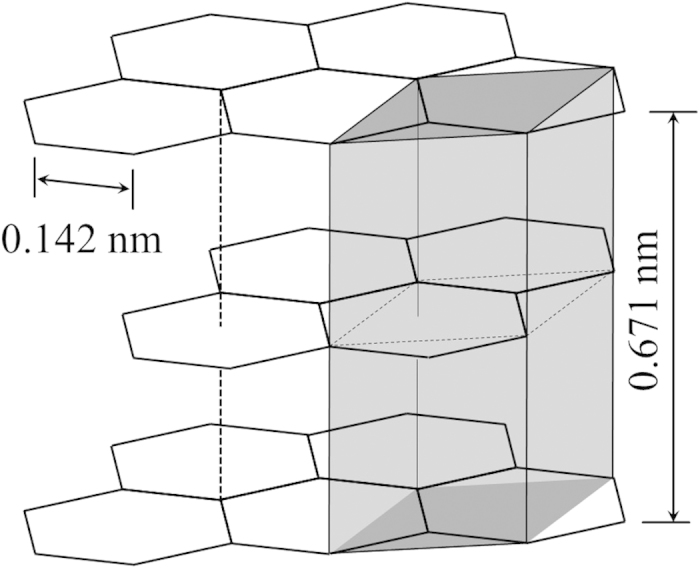
A representation of the alternating honeycomb lattice in graphite. Carbon atoms are located at intersections of thick black lines. Each carbon atom has three nearest neighbors located 0.142 nm away. The graphite unit cell is shown in the shaded gray box.

As we show in the next section, an analysis of this system based on crystallography suggests that the ion temperature is significantly higher than previously estimated. Carbon atoms in graphite are arranged in a honeycomb lattice, with alternating layers (see Fig. 1). This lattice is significantly different from the more energetically favorable configuration in which the ions are as far away from each other as possible, such as in a close-packed (CP) configuration of a face-centered cubic (FCC) or hexagonal close-packed (HCP) lattice^27^,^28^.

### Estimating the ion temperature

The kinetic energy gained by the ions in the transition from the graphite lattice to the FCC lattice can be estimated using a simple model. Before ionization occurs, the nearest-neighbor potential energy in graphite is calculated using the shortest bond length. After the plasma relaxes, the nearest-neighbor potential energy is calculated using the characteristic FCC atom separation at the same average density, neglecting compression. The effects of compression on the value of the strong coupling parameter are discussed towards the end of this article.

Calculating the change in energy from the initial graphite structure to the final FCC lattice structure requires us to know the ion charge state and electron temperature because electron screening is important. Laser “strong-field” ionization has been studied at length (see, for example, ref. 32). Given the laser intensity reported by Brown *et al.*, and considering the ionization energies of carbon^33^, the treatment in ref. 32 suggests that the ion charge state is probably closer to *Z* = 4. At solid densities, the probability of electron scattering during the laser pulse is high. Therefore the electron temperature is equal to the ponderomotive energy of a free electron in the electric field of the laser. In this case, *T*_e_ = 60 eV for a laser intensity of 10^15^ W/cm^2^. Comparing the estimated ion charge state and plasma density using averaged-atom codes, the estimated range of temperatures is 50 to 100 eV^34^. The Debye length is *λ*_D_ = (*ε*_0_*k*_B_*T*_e_/*n*_e_*e*^2^)^1/2^ = 0.085 nm, where *T*_e_ is the electron temperature and *n*_e_ is the electron density.

We now estimate the potential energy for screened C^4+^ ions in the graphite lattice and in the FCC lattice at a density of 1.14 × 10^23^ cm^−3^ (solid density). In the graphite lattice, the nearest-neighbor distance between atoms is *a*_0_ = 0.142 nm. One would expect the nearest-neighbor potential energy per atom to be


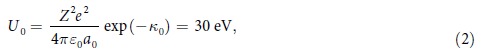


where the factor of 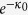 arises due to Yukawa screening, *κ*_0_ ≡ *a*_0_/*λ*_*D*_, and *Z* = 4.

In the conventional FCC lattice, the unit cell contains 4 atoms. We can denote the lattice parameter, or the side-length of the cubic unit cell, as 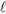. The nearest-neighbor distance between atoms, *a*_f_, is the distance from the corner of the cell to the center of the cube face, 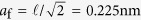 at a density of 1.14 × 10^23^ cm^−3^. The nearest-neighbor potential energy per atom in the FCC cell is


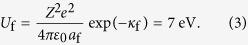


where *κ*_f_  =  *a*_f_/*λ*_*D*_. At this level of approximation, ignoring second-nearest-neighbor and other correlation effects, one would expect the ion temperature to be roughly the difference between these two energies, or approximately 23 eV. This is more than an order of magnitude higher than estimated by Brown *et al.* in ref. 29.

For this calculation, we have allowed the strongly-coupled plasma to relax to the FCC configuration^27^. The thermodynamics of strongly-coupled plasmas suggests that at low values of *κ*, the plasma might relax into a BCC configuration. However, in a strongly-screened system, with *κ* ≈ 2, the Madelung energy is lower in the FCC configuration^27^,^28^.

### CMH heating calculations

We calculate the ion temperature due to spatial structure mismatch more rigorously using molecular dynamics (MD) simulations^35^,^36^. In the simulations, the ions interact via the Yukawa potential, *u*_*ij*_(*r*) = (*Ze*/4*πε*_0_*r*_*ij*_) exp(−*r*_*ij*_/*λ*_D_), where *r*_*ij*_ is the distance between ions *i* and *j*. Although the number of particles per Debye sphere is small, first-principles calculations have shown that the Yukawa interaction can be appropriately used in this regime^37^. We initially place ions in the main simulation cell at locations corresponding to the initial crystal lattice. The equations of motion of the *N* particles are integrated using a second-order symplectic integrator (velocity-Verlet) subject to periodic boundary conditions. We find that *N* ~ 5000 ions is sufficient for the calculation to converge^22^.

In the calculation, careful attention to boundary conditions is required. Our computational grid is cubic and slightly mismatches the graphite lattice. In order to prevent this from dominating the temperature calculation, we adjust the ion positions near the boundaries to minimize edge-heating in the calculation. We also vary the size of the lattice in order to verify that this potential edge effect does not influence the result. Calculations of the ion temperature use ions located far from the simulation edges. “Movie”-like visualizations are used to verify that this systematic error is negligibly small.

The symmetry of the graphite lattice leads to exact force cancelation. Without a perturbation, no heating occurs. In our calculation, we use a variety of initial conditions to simulate a realistic laser-plasma experiment. For example, we start the simulation with the ions at zero temperature, but with their initial positions perturbed by a small amount. We also start the simulation with the ions in a perfect lattice, but with a Maxwellian velocity distribution. A range of calculations exploring these and other initial conditions show that the final ion temperature is independent of the initial perturbation.

**Figure 2:**
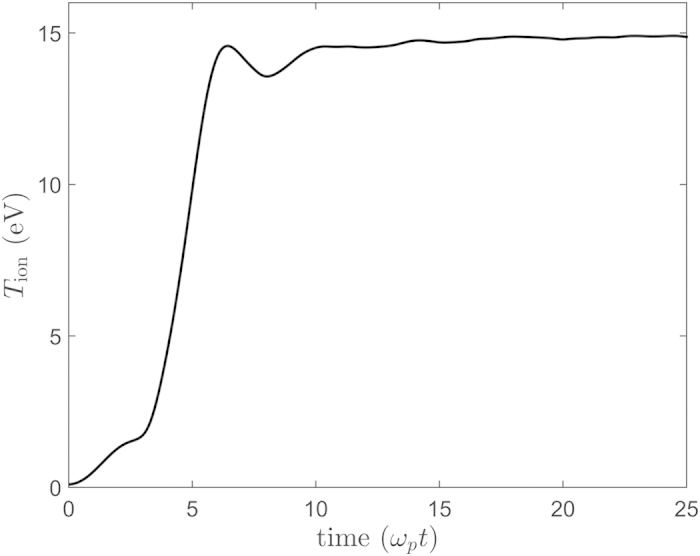
MD calculation of the ion temperature in a graphite laser-produced plasma. This simulation was started with the ions in a perfect graphite crystal, but with a velocity distribution corresponding to an ion temperature of 0.1 eV. Regardless of how the simulation is started, the ion temperature always reaches the same final temperature. This illustrates that the final temperature is determined by the mismatch between the initial graphite lattice structure and the final FCC structure of the plasma.

The result of our calculation is shown in Fig. 2. The horizontal axis is scaled using the plasma period, 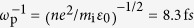. For the calculation shown, the ions are launched from a perfect lattice configuration. Their initial velocities form a Maxwellian distribution with an ion temperature of 0.1 eV – a temperature high enough that the ions start to move during the first few time steps in the calculation but small compared to the final ion temperature. The plotted quantity is the average kinetic energy per ion in the plasma. After roughly 10 plasma periods have passed, this average kinetic energy corresponds to the ion temperature. At earlier times, the ion temperature is not strictly defined because the system is out of equilibrium^18^. An initial period of slower heating is followed by a rapid temperature increase to approximately 14 eV. A modest overshoot is observed in the ion kinetic energy, as has been studied extensively in the ultracold neutral plasma community^3^,^38^. The final temperature in the plasma is within a factor of two of the estimated 23 eV temperature due to changes in the potential energy described in the simple calculation given above.

## Implications from Crystallography

Estimating the ion temperature due to crystal mismatch heating (CMH) can be more generally described. This factor-of-two calculation could be done using the atomic density and a quantity known as the atomic packing factor, 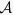. The atomic packing factor is the fraction of the crystalline unit cell that is occupied by the atoms. The very compact FCC and HCP lattices have 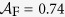. The slightly less compact body-centered cubic (BCC) lattice has 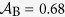. The comparatively open diamond structure has 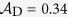. Graphite, by comparison has 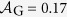.

The distance between neighboring atoms in the initial crystal lattice is twice the atomic radius, 2*r*_*a*_. In terms of atomic packing factor, this is


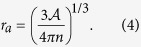


When the atoms in this lattice are ionized and move into the FCC lattice, the new separation between atoms will be similarly defined. The distance between atoms will be 2*r*_F_, with *r*_F_ = (3*A*_F_/4*πn*)^1/3^ and the density *n* equal to the density of the initial lattice.

With these definitions in mind, the difference in electric potential energy between the initial lattice and the final idealized FCC lattice can be written


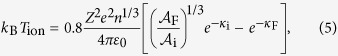


where the subscript i refers to the initial configuration and the subscript F refers to the FCC lattice. The numerical factor of 0.8 results from relating the density *n* to the distance between ions via the atomic packing factor 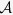. When the differences in the screening length between the initial and final configurations are not too great, this simplifies to


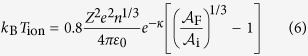


This equation could be used to give factor-of-two estimates for the CMH ion temperature in laser-produced plasmas for any of the 14 Bravais lattices. This estimate is valid for pure elements only. It could be extended to mixed-species plasmas generated from alloys or molecular solids in a straight-forward manner.

This treatment of CMH suggests that the major source of ion heating would be eliminated in laser-produced plasmas of atoms that are initially in an FCC or HCP lattice. This includes 90% of all metals and several other elements. This treatment also suggests that high ion temperatures will be generated in carbon graphite or diamond plasmas. Graphite pushers are used in laser-driven fusion work^39^. Mass effects aside, an aluminum pusher (FCC lattice) would produce a lower temperature ion plasma in the ablator. Substituting ablators of different crystallographic structures may make it possible to study the effects of ion temperature on the plasma evolution.

It may be possible to systematically explore the effects of CMH in laser-produced plasmas of the transition metals. The elements V, Cr, Mn, and Fe are all BCC at room temperature. Neighboring elements in the periodic table of Co, Ni, Cu, and Zn are all FCC or HCP. In this latter group, the effects of CMH will be absent. In the former group, CMH will be present. For an iron plasma with *Z* = 4 and *T*_e_ = 60 eV, consistent with a laser-produced plasma using a laser intensity of 10^15^ W/cm^2^, Eq. (6) suggests a CMH temperature of 0.6 eV.

In strongly-coupled plasmas, the FCC and BCC structures are thermodynamically similar. Whether one structure or the other is preferred depends on *κ*. Therefore, studying the influence of CMH using the FCC/BCC structure in metals may be challenging. Perhaps the best place to explore the effects of CMH are in carbon. Its different allotropes (graphite, diamond) give dramatically different values of the CMH. Its different geometries (bulk graphite, graphene, bucky balls, nanotubes) make it possible to explore geometric effects as well.

## Additional Considerations for Strongly-Coupled Plasmas

In Yukawa systems, where electrons screen the ion-ion interaction, the meaning of the strong coupling parameter Γ defined in Eq. (1) is debatable because it is technically valid for a one-component-plasma. However, the potential energy is more complicated than this equation suggests, and tacking a screening factor like *e*^−*κ*^ onto Eq. (1) also fails to capture the essential physics^40^. If the determination of strong coupling is based on long-range ordering in the ion system, expressions have been derived that predict the electron and ion temperatures and densities needed to achieve a given value of the pair distribution function^41^.

The value of the strong-coupling parameter is influenced by plasma compression. Adiabatic compression can occur in solid-density laser-produced plasmas. Plasmas in this regime behave as an ideal gas^42^. Adiabatic compression of an ideal gas leads to the expression *Tn*^−2/3^ = constant. Given that Eq. (1) shows that Γ*n*^1/3^ = constant in a strongly coupled plasma, adiabatic compression predicts that Γ ∝ *n*^−1^. As the density increases at constant *Z*, Γ decreases. For highly compressed plasmas, the increase of the average ionization state with density also influences Γ^43^.

Two additional heating mechanisms need to be considered in dense systems. Electron-ion thermalization heats the ions, reducing Γ further. For carbon at a density of 10^23^ cm^−3^ and an electron temperature of 60 eV, the electron-ion thermalization time is 2 ps^44^. In this short time, the electrons will heat the ions, reducing Γ even further. Three-body recombination and electron-Rydberg scattering heats the electrons and eventually also the ions^45^,^46^. The three-body recombination time in this system is less than 1 ps. Experiments in UNPs indicate that recombination occurs rapidly in strongly-coupled plasmas^47^, and this effect also needs to be considered in estimates of the time-evolving electron and ion temperatures in these systems.

All these considerations suggest that an experiment designed to explore the effects of strong coupling needs to meet several criteria. The plasma needs to be generated from a close-packed crystal. This will eliminate the influence of CMH. Heating due to electron-ion thermalization and three-body recombination can be minimized if the plasma expands before the electrons and ions reach a global equilibrium and before significant recombination has occurred^48^. The expansion velocity is *v*_exp_ = (*k*_B_*T*_*e*_/*m*_i_)^1/2^ = 10,000 m/s for Fe ions in a plasma with an electron temperature of 60 eV. Given a three-body recombination time of ~1 ps, an initial plasma size should be small enough so that the plasma doubles in size in just 1 ps, or *r*_0_ = (1 ps) (10,000 m/s) = 10 nm. Alternatively, a larger plasma could be studied if fs-laser-based temperature diagnostics were used to evaluate the ion temperature in the sub-ps temperature regime.

## Conclusion

We have shown that the initial crystal configuration of a solid can limit the maximum possible value of Γ in a laser-produced plasma. We have shown how to estimate and accurately calculate the ion temperature resulting from crystal-mismatch heating (CMH). At early enough times, before the ions have been heated by collisions with the electrons and before significant recombination has occurred but after several ion plasma periods, the ion temperature will be determined solely by CMH. In this regime, which is analogous to the many experiments in ultracold neutral plasmas, the value of the strong coupling parameter Γ should be high enough to observe Wigner crystallization in the plasma state.

## Additional Information

**How to cite this article**: Lyon, M. *et al.* Strongly-coupled plasmas formed from laser-heated solids. *Sci. Rep.* **5**, 15693; doi: 10.1038/srep15693 (2015).
